# SP600125 Induces Src and Type I IGF Receptor Phosphorylation Independent of JNK

**DOI:** 10.3390/ijms150916246

**Published:** 2014-09-15

**Authors:** Qingbin Kong, Hui Hua, Anguo Cui, Ting Shao, Peiying Song, Yangfu Jiang

**Affiliations:** 1 State Key Laboratory of Biotherapy, Section of Oncogene, West China Hospital, Sichuan University, Chengdu 610041, China; E-Mails: kongqingbincdut@163.com (Q.K.); cuianguo1618@126.com (A.C.); shaoting89410@126.com (T.S.); lanringsong@163.com (P.S.); jyangfu@scu.edu.cn (Y.J.); 2 Laboratory of Stem Cell Biology, West China Hospital, Sichuan University, Chengdu 610041, China

**Keywords:** SP600125, JNK, Src, IGF-IR

## Abstract

c-Jun *N*-terminal kinases (JNK) are members of the mitogen-activated protein kinase (MAPK) family that have important roles in signal transduction. The small molecule SP600125 is widely used in biochemical studies as a JNK inhibitor. However, recent studies indicate that SP600125 may also act independent of JNK. Here, we report that SP600125 can induce Src, type I insulin-like growth factor receptor (IGF-IR), Akt and Erk1/2 phosphorylation. Notably, these effects are independent of its inhibition of JNK. Inhibition of Src abrogates the stimulation of IGF-IR, Akt and Erk1/2 phosphorylation. IGF-IR knockdown blunts the induction of both Akt and Erk1/2 phosphorylation by SP600125. Moreover, combination of SP600125 and the Src inhibitor saracatinib synergistically inhibits cell proliferation. We conclude that SP600125 can activate Src-IGF-IR-Akt/Erk1/2 signaling pathways independent of JNK.

## 1. Introduction

c-Jun *N*-terminal kinases (JNKs) belong to the mitogen-activated protein kinase (MAPK) family, and consist of more than ten isoforms encoded by three genes, namely JNK1, JNK2 and JNK3 [1]. Phosphorylation and activation of JNK can be induced by inflammatory signals, reactive oxygen species and stress stimuli. Upon activation, JNK may regulate several important cellular processes such as cell growth, differentiation, survival and apoptosis. Downstream targets that are directly activated by JNK include c-Jun, ATF2, ELK1, SMAD4, p53 and HSF1 [2]. Previous studies have suggested that this signaling pathway contributes to a variety of physiological or pathological conditions in mammals and insects.

SP600125 is an anthrapyrazolone compound that is widely used in biochemical studies as a JNK inhibitor. In addition, analogs of SP600125 have a variety of biological activities. For example, anthrapyrazolones themselves have been investigated as anticancer agents for their DNA-binding activity [3,4]. Moreover, intensive studies have been done to investigate the effect of SP600125 on endoreplication-related apoptosis [5,6,7]. Recent studies also demonstrate that SP600125 can act as anti-inflammatory agents by modulating the expression of inflammatory factors through blocking the activation of JNK and c-Jun [8,9,10].

Despite SP600125 has been considered as a JNK inhibitor in a number of studies, recent studies demonstrate that it may also act in JNK-independent manner. For example, the ability of SP600125 to up-regulate REDD1 expression and inhibit voltage-dependent potassium ion channel is JNK-independent [11,12]. The effect of SP600125 on endoreplication results from suppressing Cdk1 activity rather than inhibiting JNK [5]. Although SP600125 reportedly induces Akt phosphorylation [7], the mechanisms underlying this effect remain unclear. Here, we show that SP600125 induces Akt and Erk1/2 phosphorylation through Src-mediated type I insulin-like growth factor receptor (IGF-IR) activation independent of JNK.

## 2. Results and Discussion

### 2.1. SP600125 Induces Type I Insulin-Like Growth Factor Receptor (IGF-IR), Akt and Erk1/2 Phosphorylation

**Figure 1:**
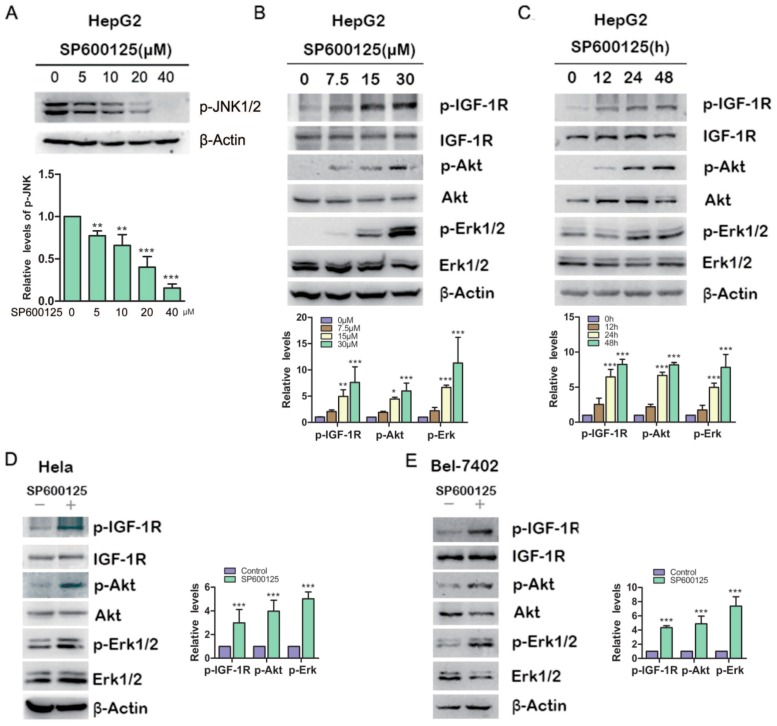
SP600125 induces type I insulin-like growth factor receptor (IGF-IR), Akt, Erk1/2 phosphorylation. (**A**) HepG2 cells were treated with increasing doses of SP600125 for 48 h, followed by western blot analysis of c-Jun *N*-terminal kinases (JNK) phosphorylation; (**B**) HepG2 cells were treated with SP600125 for 48 h at the indicated doses, followed by western blot analysis of phosphorylated IGF-1R, Akt, Erk1/2 and total IGF-1R, Akt, Erk1/2; (**C**) HepG2 cells were treated with 15 µM SP600125 for indicated periods, followed by western blot analysis of phosphorylated IGF-1R, Akt, Erk1/2 and total IGF-1R, Akt, Erk1/2; and (**D**,**E**) Hela and BEL-7402 cells were treated with 15 µM SP600125 for 48 h, followed by western blot analysis of phosphorylated IGF-1R, Akt, Erk1/2 and total IGF-1R, Akt, Erk1/2. The blots were subjected to densitometric analysis and relative quantification. Levels in SP600125-untreated cells were set as 1. A representative of three experiments was shown. *, *p* < 0.05; **, *p* < 0.01; ***, *p* < 0.001, compared with control samples.

To investigate the effects of SP600125 on Akt or Erk1/2 phosphorylation, HepG2 hepatoma cells were treated with different doses of SP600125 for 48 h, followed by western blot analysis of the levels of Akt and Erk1/2 phosphorylation. Treatment with SP600125 inhibited JNK phosphorylation and induced both Akt and Erk1/2 phosphorylaion in a dose-dependent manner (Figure 1A,B). Moreover, Akt and Erk1/2 phosphorylation was stimulated by SP600125 in a time-dependent manner (Figure 1C). Similar results were observed in BEL-7402 hepatoma cells and Hela cervical cancer cells (Figure 1D,E). These data demonstrate that SP60125 can induce Akt and Erk1/2 phosphorylation. Akt, also known as protein Kinase B (PKB), is a serine/threonine protein kinase that plays a key role in multiple cellular processes such as glucose metabolism, apoptosis, cell proliferation and migration. Akt may be activated by PI3-kinase-dependent or PI3K-independent manner [13]. In addition, Erk, the classical mitogen-activated protein kinase that is usually activated by Ras GTP-binding protein, is important intracellular signaling molecule regulating meiosis, mitosis, and postmitotic functions [14]. Erk can activate many transcription factors and downstream protein kinases [15]. Aberration in the Erk pathway is common in cancers, especially Ras, c-Raf and receptors such as EGFR and Her2. Thus, SP600125 may have important functions in signal transduction through regulating Akt and Erk1/2.

Both Akt and Erk1/2 are classical effectors downstream growth factor receptors including IGF-IR. We found that treatment with SP600125 also increased IGF-1R phosphorylation in dose-dependent and time-dependent manner in various cancer cell lines (Figure 1). In addition, we found that SP600125 from another source, Sigma, had similar effects (data not shown). IGF-IR is well known as a positive regulator of a wide range of cellular processes, ranging from cellular proliferation, migration and survival. Upon IGF stimulation, IGF-IR is activated and then recruits insulin receptor substrates (IRS) such as IRS1 and IRS2, leading to the activation of multiple signaling cascades including PI3K/Akt and MAPK pathways [16]. Although it has been reported that SP600125 can induce Akt phosphorylation [7], it is unclear whether IGF-IR mediates this effect.

### 2.2. SP600125 Activates Akt and Erk via IGF-1R Activation

**Figure 2:**
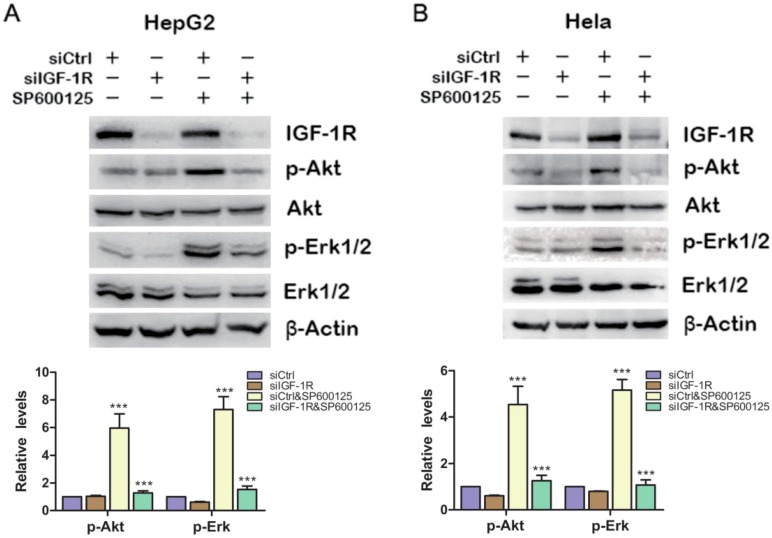
IGF-1R is required for the induction of Akt and Erk1/2 phosphorylation by SP600125. HepG2 (**A**) or Hela (**B**) cells were transfected with negative siRNA (siCtrl) or siRNA against IGF-1R for 24 h, followed by treatment with or without 15 µM SP600125 for 48 h. Total protein extracts were harvested and subjected to western blot analysis of total IGF-1R, Akt, Erk1/2 and phosphorylated Akt, Erk1/2. β-Actin was detected as loading control. The blots were subjected to densitometric analysis and relative quantification. Levels in control samples were set as 1. A representative of three experiments was shown. ***, *p* < 0.001, compared with control samples.

Because plenty of studies show that IGF-1R plays a pivotal role in regulating PI3K/Akt and Ras/MAPK signaling pathways [16], we wonder whether the effect of SP600125 on Akt and Erk1/2 phosphorylation results from IGF-IR phosphorylation. To this end, HepG2 cells were transfected with siCtrl or siRNA against IGF-1R and treated with or without SP600125. SP600125 failed to induce Akt and Erk1/2 phosphorylation upon IGF-1R knockdown (Figure 2A). Similar results were also observed in Hela cells (Figure 2B). These results indicate that the effect of SP600125 on Akt and Erk1/2 phosphorylation is downstream of IGF-1R.

### 2.3. Effects of SP600125 on IGF-1R Signaling Pathway Is Independent of c-Jun N-Terminal Kinases (JNK)

**Figure 3:**
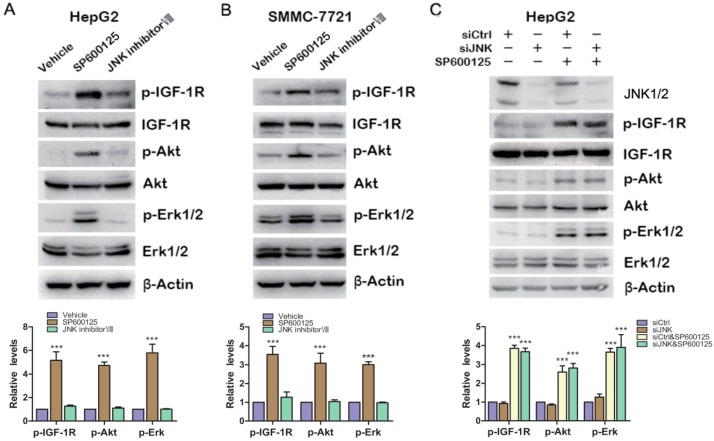
Effects of SP600125 on IGF-1R signaling pathway is independent of JNK. (**A**,**B**) HepG2 and SMMC-7721 cells were treated with 15 µM SP600125 or 20 µM JNK inhibitor VIII for 48 h. Total protein extracts were harvested and subjected to western blot analysis of phosphorylated IGF-1R, Akt, Erk1/2 and total IGF-1R, Akt, Erk1/2; and (**C**) HepG2 cells were transfected with siCtrl or siRNA against JNK for 24 h, followed by treatment with or without 15 µM SP600125 for 48 h. Total protein extracts were harvested and subjected to western blot analysis of phosphorylated IGF-1R, Akt, Erk1/2 and total IGF-1R, Akt, Erk1/2. β-Actin was detected as loading control. The blots were subjected to densitometric analysis and relative quantification. Levels in control samples were set as 1. A representative of three experiments was shown. ***, *p* < 0.001, compared with other groups.

As we know, SP600125 is widely used as JNK inhibitor. To determine whether JNK is involved in SP600125-induced IGF-1R signaling, HepG2 cells and SMMC-7721 cells were treated with SP600125 or JNK inhibitor VIII, another specific JNK inhibitor. SP600125 induced IGF-1R, Akt and Erk1/2 phosphorylation in both HepG2 and SMMC-7721 cells (Figure 3A,B). JNK inhibitor VIII slightly induced IGF-1R phosphorylation, while it did not induce Akt and Erk1/2 phosphorylation (Figure 3A,B). In addition, HepG2 cells were transfected with sictrl or siRNA against JNK1/2 and treated with or without SP600125.Western blot analysis showed that JNK1/2 was efficiently knocked down. Knockdown of JNK1/2 alone had no effect on IGF-1R signaling cascades (Figure 3C). In addition, SP600125 induced IGF-1R signaling both in the absence or presence of JNK1/2 (Figure 3C). These data suggest that SP600125 may exert its effects on IGF-1R signaling pathway independent of JNK1/2.

### 2.4. Src Is Involved in SP600125-Induced IGF-1R Phosphorylation

**Figure 4:**
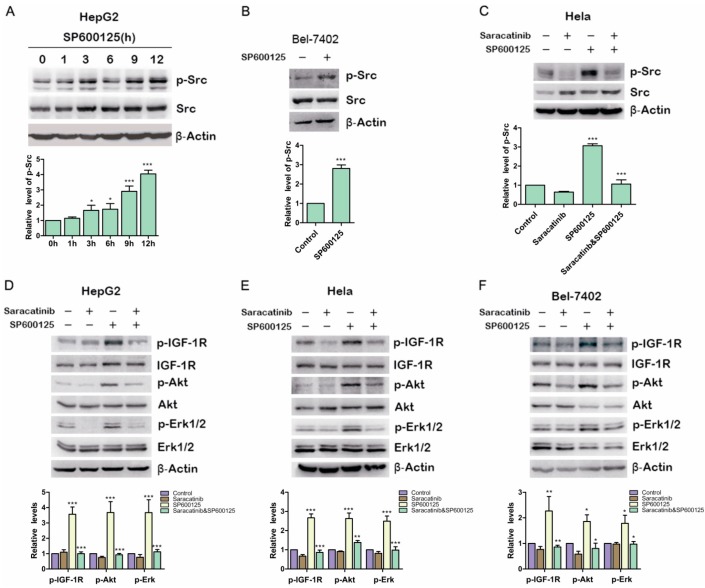
Src is involved in SP600125-induced IGF-1R phosphorylation. (**A**) HepG2 cells were treated with 15 µM SP600125 for the indicated time. Total protein extracts were harvested and subjected to western blot analysis of phosphorylated Src and total Src; (**B**) Bel-7402 cells were treated with 15 µM SP600125 for 48 h. Total protein extracts were harvested and subjected to western blot analysis of phosphorylated Src and total Src; (**C**) Hela cells were pretreated with 5 µM saracatinib for 3 h and then treated with or without 15 µM SP600125 for 48 h. Total protein extracts were harvested and subjected to western blot analysis of phosphorylated Src and total Src; and (**D**–**F**) HepG2, Hela and Bel-7402 cells were pretreated with 5 µM saracatinib for 3 h and then treated with or without 15 µM SP600125 for 48 h. Total protein extracts were harvested and subjected to western blot analysis of phosphorylated IGF-1R, Akt, Erk1/2 and total IGF-1R, Akt, Erk1/2. β-Actin was detected as loading control. The blots were subjected to densitometric analysis and relative quantification. Levels in control samples were set as 1. A representative of three experiments was shown. *, *p* < 0.05; **, *p* < 0.01; ***, *p* < 0.001, compared with control samples.

Previous studies showed that IGF-1R can be phosphorylated and then activated by Src [17] Therefore, we examined if SP600125 could induce Src phosphorylation. HepG2 cells were treated with SP600125 for different time points. Src phosphorylation was up-regulated significantly at 12 h after SP600125 treatment (Figure 4A). Similar results were obtained in BELl-7402 cells (Figure 4B). To determine whether Src activity blockade can inhibit the induction of IGF-1R phosphorylation by SP600125, HepG2 cells and Hela cells were pretreated with or without saracatinib, an Src inhibitor, and then treated with or without SP600125. Saracatinib not only blocked SP600125-induced Src phosphorylation but also inhibited the effects of SP600125 on IGF-1R signaling cascades (Figure 4C–F). These results suggest that SP600125-induced IGF-1R phosphorylation is mediated by Src. Src is a non-receptor tyrosine kinase that plays an important role in diverse cellular processes including proliferation, survival, angiogenesis and metastasis. Reciprocal regulation between Src and receptor tyrosine kinases has been intensively studied. Src may cooperate with receptor tyrosine kinase to perform its role [18]. The activation of Src is commonly correlated with a variety of tumors [19]. It has been reported that SP600125 induce cell cycle arrest by up-regulating p21 activity and expression. This effect is mediated by Erk/Sp1 and PI3K/Akt pathways. Inhibition of PI3K/Akt can sensitize SP600125-induced apoptosis [7].

### 2.5. Combination of SP600125 and Saracatinib Synergistically Inhibits Cell Proliferation

**Figure 5:**
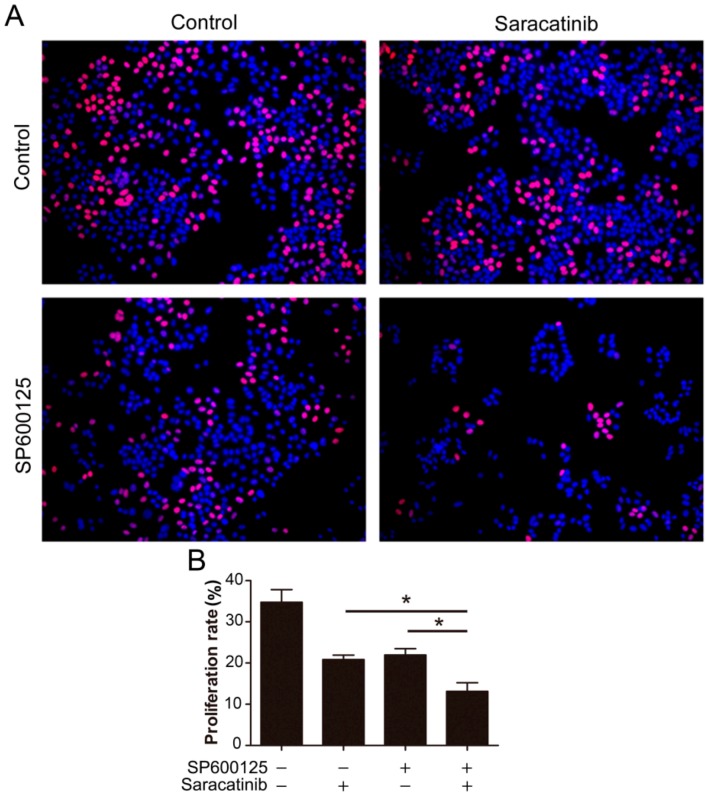
Combination of SP600125 and saracatinib synergistically inhibits HepG2 cell proliferation. (**A**) HepG2 cells were pretreated with 5 µM SP600125 for 3 h and then treated with or without 10 µM SP600125 for 48 h, followed by EdU labeling. A representative of two independent experiments in triplicate is shown; and (**B**) The proliferation rate was plotted. Values represent mean ± SD (*n* = 3). *, *p* < 0.05.

Given that SP600125 induces Src phosphorylation, we tested if inhibition of Src can potentiate the anti-cancer activity of SP600125. We utilized EdU assays to detect the effect of combination of saracatinib and SP600125 on cell proliferation. While both saracatinib and SP600125 slightly inhibited HepG2 cell proliferation, combination of saracatinib and SP600125 significantly inhibited cell proliferation (Figure 5). These data suggest that activation of Src by SP600125 may compromise the inhibitory effect of SP600125 on cell proliferation. Also, treatment of tumor cells with SP600125 may sensitize them to Src inhibitors.

Previous studies demonstrate that SP600125 may potently inhibit voltage-dependent potassium ion channel and up-regulate REDD1 in JNK-independent manner [11,12]. G iven that SP600125 is used as a JNK blocker in a number of studies of the roles of JNK in signal transduction, these studies may be of importance and primary interest. Interpreting the results based on SP600125 as a JNK inhibitor should be done with extreme care. Other JNK inhibitors such as inhibitor VIII, which does not induce Akt and Erk1/2 phosphorylation, may be used in future studies on JNK to determine roles of JNK in cellular processes, rather than SP600125. Notably, JNK inhibitor VIII has been shown not to cause cellular growth arrest [20]. Future studies are warranted to determine how SP600125 induces Src phosphorylation.

In summary, this study uncovers that SP600125 activates Src-IGF-1R-Akt/Erk pathways independent of inhibition of JNK. While JNK appears to be an attractive target for treating cancer, especially hepatocellular carcinoma [21], activation of Src-IGF-1R-Akt/Erk pathways may compromise the anti-cancer effect of SP600125. Combination of SP600125 with Src/IGF-1R inhibitor may be a strategy for cancer treatment.

## 3. Materials and Methods

### 3.1. Reagents

SP600125 was purchased from Beyotime Institute of Biotechnology and Sigma. JNK inhibitor VIII was purchased from Merck (Darmstadt, Germany). Saracatinib was purchased from LC Laboratories. Antibodies for p-IGF-1R (Y1135) (#2182-1) were purchased from Epitomics (Burlingame, CA, USA). Antibodies for IGF-1R (#3027), p-Akt (S473) (#4060), Akt (#3063), p-Erk1/2 (T202/Y204) (#4370S), Erk1/2 (#4695), p-Src (Y416) (#6943P), Src (#2123), p-JNK (T183/Y185) (#9251), JNK (#9258P) were purchased from Cell Signaling Technology (Beverly, MA, USA). Antibody for β-actin (#KM9001) was purchased from Tianjin Sungene Biotech Co., Ltd., Tianjin, China.

### 3.2. Cell Culture

Hepatoma cell lines HepG2, SMMC-7721, Bel-7402 and cervical cancer cell line Hela were all obtained from Cell Lines Bank, Chinese Academy of Science (Shanghai, China). The cells were maintained in Dulbecco Minimal Essential Medium (DMEM) containing 10% fetal bovine serum and 50 units/mL of penicillin and 50 µg/mL streptomycin sulfate, and incubated at 37 °C in a humidified atmosphere of 5% CO_2_.

### 3.3. RNA Interference

All siRNAs were custom-synthesized products of Ribobio Co., Ltd. (Guangzhou, China). The target sequence of siIGF-1R is CAATGAGTACAACTACCGC. The target sequence of siJNK1/2 is GAAAGAATGTCCTACCTTC. The negative siRNA (siCtr) was provided by Ribobio Co., Ltd. (Guangzhou, China). The double-stranded siRNA was dissolved in DEPC-treated water. Subconfluent proliferating cells were transfected with Lipofectamine 2000 (Invitrogen, Carlsbad, CA, USA) according to the instructions of manufacturer. The final concentration of siRNAs was 50 nM.

### 3.4. Western Blotting

Total protein extracts were harvested by lysis with RIPA buffer (50 mM Tris–HCl, 150 mM NaCl, 1 mM EDTA, 1% NP-40, 0.25% Na-deoxycholate, 1 mM PMSF, 1 mM NaVO_3_, 1 mM NaF). Protein concentrations were quantified by BCA protein assay (Thermo Scientific Inc., Rockford, IL, USA). Samples containing 30 μg of protein were resolved by SDS-PAGE and transferred to PVDF membrane (Millipore Corporation, Billerica, MA, USA). Membranes were incubated with primary antibodies overnight at 4 °C and appropriate HRP-secondary antibodies for 1 h at room temperature. Detection was performed with chemiluminescent agents (Beyotime, Haimen, China). Images were gathered by Alpha Innotech’s FluorChem imaging system (Alpha Innotech Corp., San Leandro, CA, USA). Densitometric analysis was performed with Adobe Photoshop (San Jose, CA, USA).

### 3.5. Cell Proliferation Assay

HepG2 cells were plated in 96-well plate at 3500 cells per well. Cells were treated with SP600125 or saracatinib or both. 48 h later, the cells were incubated with 50 μM EdU (#C10310-1) (Ribobio, Guangzhou, China) medium for 2 h. Then the cells were treated with 4% Polyoxymethylene for 30 min, 2 mg/mL glycine for 10 min, and incubated with 0.5% TritonX-100 for 10 min. The cells were incubated in Apollo^®^ 567 for 30 min at room temperature avoiding light, washed with PBS, and then incubated with Hoechst 33342 for 15 min at room temperature avoiding light. After washing the cells with PBS three times, images were gathered by fluorescence microscope.

### 3.6. Statistical Analysis

All statistical analysis was performed with one-way ANOVA. All statistical tests were two-tailed, and difference to be considered to be statistically significant when *p* < 0.05.

## 4. Conclusions

The current study demonstrates that SP600125 activates Src-IGF-IR-Akt/Erk1/2 pathways in a JNK-independent manner.
